# The Role of Telehealth in Promoting Equitable Abortion Access in the United States: Spatial Analysis

**DOI:** 10.2196/45671

**Published:** 2023-11-07

**Authors:** Leah R Koenig, Andréa Becker, Jennifer Ko, Ushma D Upadhyay

**Affiliations:** 1 Advancing New Standards in Reproductive Health Department of Obstetrics, Gynecology & Reproductive Sciences University of California, San Francisco San Francisco, CA United States; 2 Department of Epidemiology and Biostatistics University of California, San Francisco San Francisco, CA United States; 3 Center for Gender and Health Justice University of California Global Health Institute Oakland, CA United States

**Keywords:** telehealth, abortion, spatial analysis, health equity, barriers, abortion access, legal, health equity, young people, remote, rural

## Abstract

**Background:**

Even preceding the Supreme Court’s 2022 *Dobbs v. Jackson Women’s Health Organization* decision, patients in the United States faced exceptional barriers to reach abortion providers. Abortion restrictions disproportionately limited abortion access among people of color, young people, and those living on low incomes. Presently, clinics in states where abortion remains legal are experiencing an influx of out-of-state patients and wait times for in-person appointments are increasing. Direct-to-patient telehealth for abortion care has expanded since its introduction in the United States in 2020. However, the role of this telehealth model in addressing geographic barriers to and inequities in abortion access remains unclear.

**Objective:**

We sought to examine the amount of travel that patients averted by using telehealth for abortion care, and the role of telehealth in mitigating inequities in abortion access by race or ethnicity, age, pregnancy duration, socioeconomic status, rural residence, and distance to a facility.

**Methods:**

We used geospatial analyses and data from patients in the California Home Abortion by Telehealth Study, residing in 31 states and Washington DC, who obtained telehealth abortion care at 1 of 3 virtual abortion clinics. We used patients’ residential ZIP code data and data from US abortion facility locations to document the round-trip driving distance in miles, driving time, and public transit time to the nearest abortion facility that patients averted by using telehealth abortion services from April 2021 to January 2022, before the *Dobbs* decision. We used binomial regression to assess whether patients reported that telehealth was more likely to make it possible to access a timely abortion among patients of color, those experiencing food insecurity, younger patients, those with longer pregnancy durations, rural patients, and those residing further from their closest abortion facility.

**Results:**

The 6027 patients averted a median of 10 (IQR 5-26) miles and 25 (IQR 14-46) minutes of round-trip driving, and 1 hour 25 minutes (IQR 46 minutes to 2 hours 30 minutes) of round-trip public transit time. Among a subsample of 1586 patients surveyed, 43% (n=683) reported that telehealth made it possible to obtain timely abortion care. Telehealth was most likely to make it possible to have a timely abortion for younger patients (prevalence ratio [PR] 1.4, 95% CI 1.2-1.6) for patients younger than 25 years of age compared to those 35 years of age or older), rural patients (PR 1.4, 95% CI 1.2-1.6), those experiencing food insecurity (PR 1.3, 95% CI 1.1-1.4), and those who averted over 100 miles of driving to their closest abortion facility (PR 1.6, 95% CI 1.3-1.9).

**Conclusions:**

These findings support the role of telehealth in reducing abortion-related travel barriers in states where abortion remains legal, especially among patient populations who already face structural barriers to abortion care. Restrictions on telehealth abortion threaten health equity.

## Introduction

Distance has long been understood to be a key barrier to abortion access in the United States. The distance that patients must travel to the nearest abortion provider has increased substantially in the wake of the July 2022 US Supreme Court’s *Dobbs v. Jackson Women’s Health Services* decision, which has led to abortion being banned in at least 14 states [1,2]. As a result, providers in protected access states—states where abortion care remains legally available—have experienced an influx of out-of-state patients, which is increasing wait times to care, and may force abortion seekers to travel farther than they would if they could obtain care at the facility closest to their home [3].

Prior studies have found that the further a patient must travel to a provider, the less likely they are to obtain a desired abortion [4,5]. National data indicate that longer travel distances to care due to a spike in antiabortion restrictions in the United States in recent years have contributed to the decreased abortion rate [6]. As of 2019, 20% of people who can get pregnant in the United States lived 43 or more miles from an abortion provider [7] and as of 2018, the United States had 27 “abortion deserts,” that is, major cities with no abortion provider within 100 miles [8]. These geographic barriers to abortion facilities exist even within states where abortion is legal [9].

Research has also demonstrated that the impacts of state abortion bans are experienced unequally. Increased distance poses particular challenges for low-income patients due to the lower likelihood of car ownership, loss of wages from the time needed off work, transportation costs for gas or transit fare, as well as the cost of lodging and childcare [10]. People living in rural areas, the South, Midwest, and Mountain West; Black and Hispanic individuals; and minors face greater distances to reach an abortion provider [11,12]. Since the *Dobbs* decision, travel times to abortion facilities have increased disproportionately for Black and Indigenous populations [2].

However, direct-to-patient telehealth abortion may greatly mitigate existing geospatial inequities in abortion access [10,13], despite only being legal in 24 states and Washington, DC as of September 2023 (although more states permit abortion care but prohibit telehealth) [14,15]. In direct-to-patient telehealth abortion, a clinician can interact with patients remotely through videoconferencing or secure messaging and dispense abortion medications via mail-order pharmacies. Prior to 2020, telehealth for abortion was limited to clinic-to-clinic models with remote patient-provider interactions that took place at medical facilities with ultrasonography and other in-person tests [16-20]. Early in the COVID-19 pandemic, professional organizations including The American College of Obstetricians and Gynecologists endorsed a telehealth abortion model [21]. Recent studies have demonstrated the safety and effectiveness of this model of abortion care that can take place without any visits to a clinic or medical facility [22-25]. However, existing research has not captured the travel averted due to telehealth access.

In addition to the potential benefits of direct-to-patient telehealth (subsequently referred to as telehealth) in reducing abortion-related travel, the role of these models in enabling people to obtain a wanted, timely abortion is not well understood. A study conducted in the Pacific Northwest found that patients who used telehealth abortion services resided further from an abortion facility than those who accessed in-clinic care and that some patients chose telehealth even when a clinic is geographically convenient, indicating a preference for telehealth among some patients who obtained an abortion [26]. It is critical to understand who benefits most from abortion models that allow patients to carry out the entire abortion process from home. Few studies have examined the extent to which patients choose telehealth abortion when they have access to in-clinic abortion or whether telehealth is making an otherwise inaccessible abortion possible, therefore reducing barriers to access.

Therefore, we aimed to estimate the amount of travel patients avert by using a telehealth service for medication abortion among a sample of patients who obtained abortions who overwhelmingly resided in states that allow telehealth for abortion care. We also aimed to understand the role that telehealth plays in enabling people to obtain a timely abortion, especially for those who face the greatest barriers to in-clinic care.

## Methods

### Overview

We used data from the California Home Abortion by Telehealth (CHAT) Study, a study that examines medication abortions provided via telehealth from 3 US virtual clinics, defined as telehealth abortion clinics without brick-and-mortar facilities: Choix, Hey Jane, and Abortion on Demand. While the study was initiated in California, it expanded as the virtual clinics expanded their services to 20 states and Washington, DC. We obtained anonymized clinical chart data from patients who obtained abortion care from these clinics for a defined period between April 2021 and January 2022. At the time of consent to care with the virtual clinic, patients provided permission for the telehealth provider to share their anonymized clinical records with researchers in accordance with each clinic’s privacy policy. All clinical data received were deidentified apart from ZIP code (United States postal codes) and dates of service.

In addition, each virtual clinic invited patients who were approved for abortion care during a defined period between June 2021 and January 2022 to participate in a series of 3 surveys on the abortion provider’s telehealth platform. Directly following the abortion intake, patients were directed to a page containing detailed information about the study and, if interested, patients provided electronic informed consent prior to their participation. The first survey was administered directly following abortion intake, after obtaining informed consent. Two follow-up surveys were administered within 4 weeks after abortion intake. All surveys were completed on the virtual clinics’ telehealth platforms to maximize continuity and survey completion and minimize disruption for participants. Virtual clinics continued to invite patients to complete surveys until approximately 400 patients from each completed all 3 surveys. These participant surveys were linked with their clinical records, and all data were standardized and stored on a secure Research Electronic Data Capture (REDCap; Vanderbilt University) server [27]. We excluded records for abortions where the patient did not take the abortion medications, surveys that were not matched to a clinical chart, and patients with invalid ZIP codes.

### Measures

Our key independent variables of interest reflected the amount of travel to the nearest abortion provider averted by using telehealth abortion services. Patient’s ZIP codes were recorded in both the clinical chart data and in the surveys. For cases where the clinical chart ZIP code (used for mailing the medications) differed from the ZIP code reported in the survey, we retained the survey ZIP codes with the rationale that some patients may use addresses besides their home addresses for medication delivery. Using ArcGIS (Esri), we first calculated the centroid—the center point of all coordinate points inside the ZIP code polygon—of each unique ZIP code of patients’ residences. Next, we used the Advancing New Standards in Reproductive Health 2021 Abortion Facility Database to establish the coordinates of publicly advertising US abortion facilities [28]. We used the ArcGIS Find Nearest feature to calculate the driving distance between the coordinates of the centroid of each patient’s ZIP code and the coordinates of the closest abortion facility in miles. We doubled the calculation of 1-way driving distance in miles to calculate the total round-trip driving distance averted in miles. This ArcGIS calculation also generated 1-way travel time in minutes without traffic, which we doubled to develop estimates of round-trip travel time averted and converted to hours. We calculated public transit travel time in hours between patients’ ZIP codes and abortion providers’ geographic coordinates using the gmapsdistance package in R (R Core Team) [29].

Our outcome of interest was a measure that reflected whether telehealth made it possible to access an abortion in a timely manner (Multimedia Appendix 1). The original item was phrased: “If you didn't have an abortion through telehealth, what would have happened?” Response options were: “I would have gotten an abortion at a clinic soon;” “I would have gotten an abortion at a clinic, but it would have been a while;” “I would have continued the pregnancy;” “I don’t know what would have happened;” or “something else.” If the patient responded with “something else,” they could provide free-text responses. These free-text responses included the following themes: patients would have attempted to self-manage their abortion (primarily seeking abortion pills through other channels), traveled out of state, or sought abortion care at a clinic without a specified time frame. We dichotomized this variable to reflect whether the patient perceived that telehealth made it possible to have an abortion in a timely manner (“I would have gotten an abortion at a clinic, but it would have been a while;” “I would have continued the pregnancy;” “I don’t know what would have happened;” or “something else”), versus not (“I would have gotten an abortion at a clinic soon”).

We also examined patient characteristics including: patient age at the time of abortion screening (younger than 18, 18-24, 25-29, 30-34, and ≥35 years); pregnancy duration (<35, 35-49, 50-62, and ≥63 days); self-reported race or ethnicity (Asian, Native Hawaiian, or Pacific Islander; Black; Hispanic or Latinx; White; multiracial, Native American, American Indian, or Alaska Native; Middle Eastern or North African; or unknown); urban versus suburban or rural residence based on the rural-urban commuting area codes corresponding to the patient’s ZIP code [30]; and the round-trip driving distance to the nearest abortion facility (<5, 5-24, 25-49, 50-99, and ≥100 miles). In regression analyses, we collapsed patient age (younger than 24, 25-29, 30-34, ≥35 years) due to small cell sizes.

### Data Analysis

We first described the median, IQR, and total amounts of round-trip driving distance, driving time, and public transit time averted using telehealth for abortion across the clinical chart sample. Next, we calculated median driving distances by the patient characteristic examined and tested for differences in driving distances by each characteristic using Kruskal-Wallis tests.

We used bivariate binomial regression to estimate prevalence ratios (PRs). We then calculated marginal estimates from these binomial regression models to estimate the proportion of participants within each category for whom telehealth made it possible to have an abortion in a timely manner as prevalence percentages (PPs). Analyses were conducted using ArcGIS Online, Stata (version 17.0; StataCorp), and RStudio (version 2022.10.0; Posit, PBC).

### Ethical Considerations

This study was approved by the University of California, San Francisco institutional review board (20-32951). Clinical chart data that were deidentified except for patients’ ZIP codes and dates of service were obtained from participating virtual clinics in accordance with their privacy policies. The subsample of patients who participated in detailed surveys provided electronic informed consent for their participation and were remunerated with a US $50 electronic debit card after the completion of the final survey. Clinical chart and survey data from participating virtual clinics were standardized and stored on a secure REDCap server [27].

## Results

### Overview

We obtained records for 6154 abortions provided by the virtual clinics between April 2021 and January 2022. Of those, 120 did not take medications, and 7 patients listed ZIP codes from which driving and transit distances could not be calculated, leaving 6027 records included overall. Among these, 1600 patients also participated in CHAT Study baseline surveys. The ZIP codes in the dataset corresponded to 31 states and Washington, DC.

### Travel Averted

**Figure 1:**
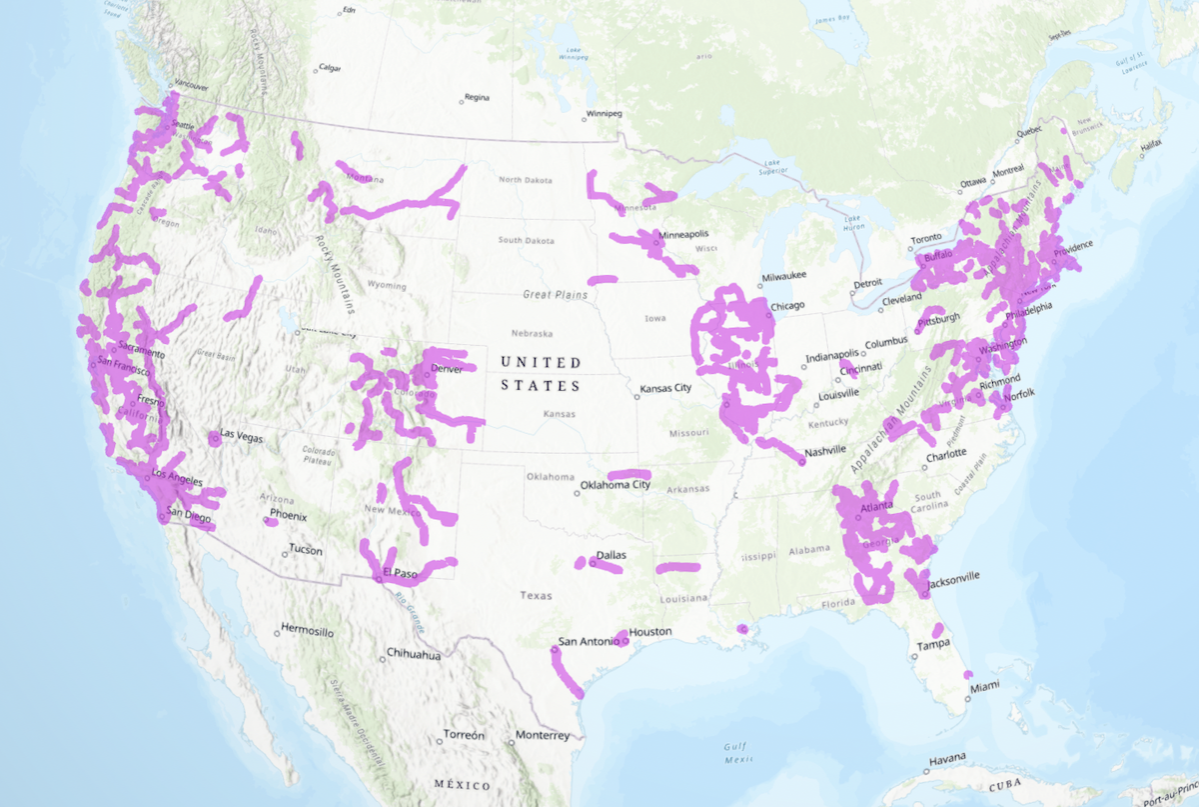
Driving routes to the closest abortion facility averted through telehealth (N=6027).

The 6027 patients in this analysis averted a median of 10 (IQR 4.5-26.0, range 0.13-566) miles of round-trip driving travel and 25 minutes (IQR 14.1-46.1 minutes, range <1 minute-9 hours) of driving time each. Driving routes averted are depicted in Figure 1 and illustrate the vast distances averted and the distribution of these routes by region. This corresponded to a total of 162,663 miles and 4195 hours of driving averted across the sample (Multimedia Appendix 2). Next, we examined the total public transit time averted for the 5116 (85%) patients for whom public transit routes were available between patients’ home ZIP codes and the nearest abortion facility. Patients in the sample averted a median of 1 hour 25 minutes (IQR 45.6 minutes-2 hours 30 minutes, range 2 minutes-48 hours) and a total of 11,720 hours of public transit time. Among the subsample of 1600 patients who completed surveys, patients averted a median of 10 (IQR 4.5-24.6) miles and 25 (IQR 14.3-44.1) minutes of driving, and 1 hour 26 minutes (IQR 46.5 minutes-2 hours 26 minutes) of public transit time. In total, this group of survey participants averted 41,746 miles and 1096 hours of driving, and 3070 hours of public transit time.

### Sample Description

Characteristics of the clinical chart sample and of the survey participants are presented in Table 1. Among the survey subsample, most patients were 25-34 (51%, n=823) years of age and had pregnancy durations between 5 and 7 weeks (55%, n=885). Over half (53%, n=841) identified as White and in the remaining sample 13% (n=203) identified as Hispanic or Latinx; 9% (n=148) identified as Black; 7% (n=105) as Asian, Native Hawaiian, or Pacific Islander; and 19% (n=303) reported more than 1 race or ethnicity, another race or ethnicity, or their race or ethnicity was unknown. In total, 91% (n=1454) of the sample resided in an urban area.

Next, we examined whether patients perceived that telehealth made it possible to have an abortion in a timely manner. Approximately one-fourth (23%, n=371) thought they would have had an abortion at a clinic, but it would have taken some time. Meanwhile, 17% (n=268) did not know what would have happened, and 2% (n=29) said they would have continued the pregnancy. The other 57% (n=903) of the sample thought they would have gotten an abortion at a clinic soon.

We then evaluated differences in travel averted by patient characteristics (Table 2). White patients averted the most driving to the nearest abortion facility (median 12, IQR 5-30 miles), while Asian, Native Hawaiian, or Pacific Islander patients averted the least (median 8, IQR 3-16 miles). Urban residents averted a median of 9 (IQR 4-19) miles of driving time, while those living in rural areas averted a median of 83 (IQR 47-141) miles. The driving miles patients averted did not differ by age, pregnancy duration, or food insecurity.

**Table 1:** Description of the clinical chart sample and survey subsample.

Factor			Clinical chart sample (n=6027), n (%)		Survey subsample (n=1600), n (%)
**Age (years)**					
	<18	30 (0.5)		8 (0.5)	
	18-24	1458 (24.2)		448 (28.0)	
	25-34	3088 (41.2)		823 (51.4)	
	≥35	1451 (24.1)		321 (20.1)	
**Pregnancy duration at abortion intake (days)**					
	<35	1736 (28.8)		448 (28.0)	
	35-49	3333 (55.3)		885 (55.3)	
	50-62	825 (13.7)		229 (14.3)	
	≥63	128 (2.1)		38 (2.4)	
**Race or ethnicity**					
	Asian, Native Hawaiian, or Pacific Islander	270 (4.5)		105 (6.6)	
	Black	413 (6.9)		148 (9.2)	
	Hispanic or Latinx	339 (5.6)		203 (12.7)	
	White	2488 (41.3)		841 (52.6)	
	Multiracial, Native American, American Indian, or Alaska Native; Middle Eastern or North African; or unknown	2517 (41.8)		303 (18.9)	
**Food insecurity in past month**					
	No	—^a^		1148 (72.8)	
	Yes	—		428 (27.2)	
**Residence**					
	Suburban or rural	593 (9.8)		146 (9.1)	
	Urban	5434 (90.2)		1454 (90.9)	
**Round-trip driving miles averted (miles)**					
	<5	1705 (28.3)		437 (27.3)	
	5-24	2765 (45.9)		775 (48.4)	
	25-49	698 (11.6)		165 (10.3)	
	50-99	467 (7.8)		120 (7.5)	
	≥100	392 (6.5)		103 (6.4)	
**If you didn’t have an abortion through telehealth, what would have happened?**					
	I would have gotten an abortion at a clinic soon	—		903 (56.9)	
	I would have gotten an abortion at a clinic, but it would have been a while	—		371 (23.4)	
	I would have continued the pregnancy	—		29 (1.8)	
	Something else	—		15 (0.9)	
	I don’t know what would have happened	—		268 (16.9)	

^a^—: not available.

**Table 2:** Round-trip driving distance averted, by patient characteristics (N=1600).

		Driving miles averted		
		Median (IQR)	*P* value	
**Age (years)**			.54	
	<18	14.5 (5.1-46.2)		
	18-24	9.5 (4.0-24.3)		
	25-34	10.2 (4.4-24.0)		
	≥35	11.4 (5.1-25.6)		
**Pregnancy duration at abortion intake (day)**			.12	
	<35	11.1 (4.7-25.0)		
	35-49	10.3 (4.6-23.4)		
	50-62	8.8 (3.7-24.5)		
	≥63	16.0 (6.3-57.5)		
**Race or ethnicity**				.001
	Asian, Native Hawaiian, or Pacific Islander	7.7 (3.4-15.6)		
	Black	9.8 (4.9-21.5)		
	Hispanic or Latinx	8.3 (5.0-19.3)		
	White	12.0 (4.7-30.2)		
	Multiracial, Native American, American Indian, Alaska Native, Middle Eastern or North African, or unknown	9.2 (4.4-19.4)		
**Food insecurity in past month**				.96
	No	10.3 (4.6-23.6)		
	Yes	10.2 (4.5-25.5)		
**Residence**				<.001
	Urban	9.3 (4.3-19.4)		
	Suburban or rural	82.6 (47.2-140.7)		

### The Role of Telehealth in Abortion Access

**Figure 2:**
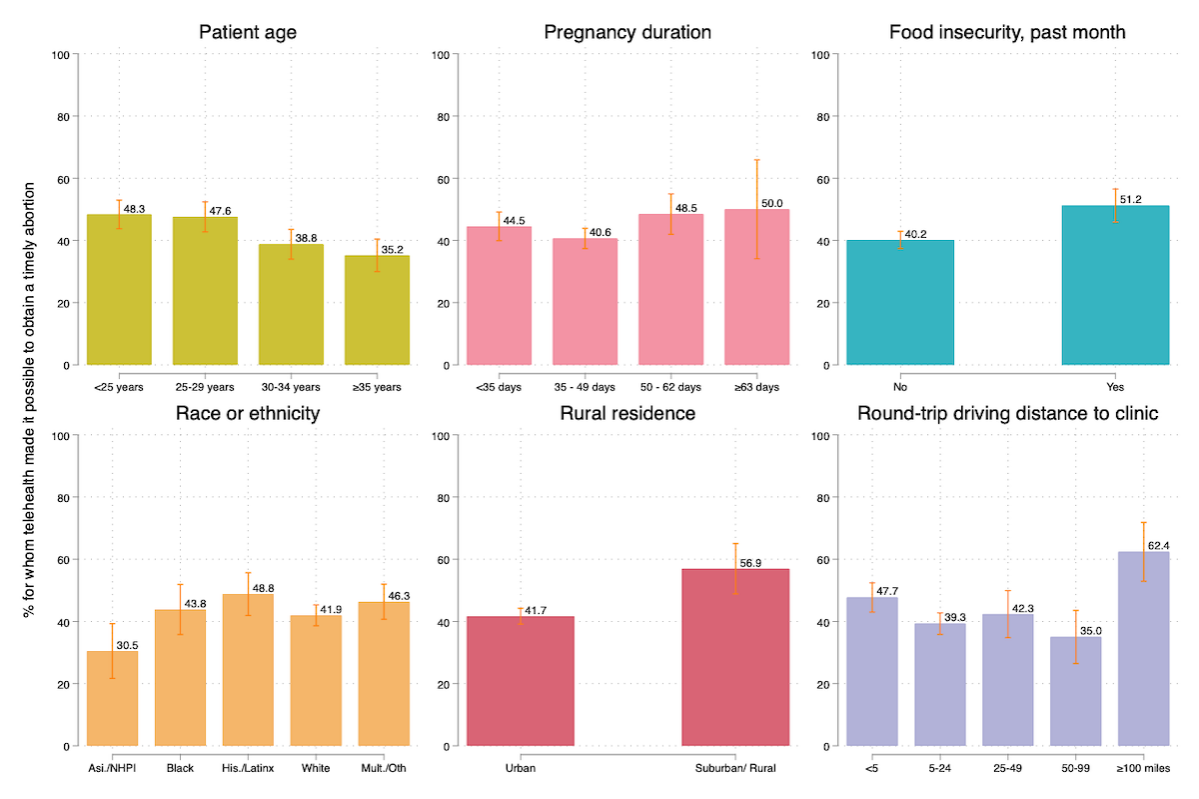
Marginal estimates of the proportion for whom telehealth made it possible to obtain a timely abortion, by patient characteristics, calculated from multivariable binomial regression (N=1586). NHPI: Native Hawaiian or Pacific Islander.

Next, we examined the differences in whether telehealth made it possible to obtain an abortion in a timely manner by patient characteristics and by the amount of travel averted (Table 3 and Figure 2). Compared to patients who were 35 years of age or older (PP 35.2%), telehealth was more likely to make abortion possible for patients 24 years of age or younger (PP 48.3%, PR 1.4, 95% CI 1.2-1.6) and for those of 25-29 years of age (PP 47.6%, PR 1.4, 95% CI 1.1-1.6). Patients who experienced food insecurity in the past month were more likely than those who had not experienced food insecurity to have reported that telehealth made a timely abortion possible (PP 51.2% vs 40.2%, PR 1.3, 95% CI 1.1-1.4). When we examined differences by race or ethnicity, patients who were Asian, Native Hawaiian, or Pacific Islander were less likely to report that telehealth made it possible to obtain a timely abortion than White patients (PP 30.5% vs 41.5%, PR 0.7, 95% CI 0.5-1.0). For patients who lived in rural areas, telehealth was more likely to make timely abortion care possible than those who resided in urban areas (PP 56.9% vs 41.7%, PR 1.4, 95% CI 1.2-1.6). Compared to those who averted 5-25 miles of driving to reach an abortion provider (PP 39.3%), those who averted less than 5 miles of round-trip driving (PP 47.7%, PR 1.2, 95% CI 1.1-1.4) or more than 100 miles of round-trip driving (PP 62.4%, PR 1.6, 95% CI 1.3-1.9) were more likely to perceive that telehealth made a timely abortion possible than those that averted less travel. The proportion of patients for whom telehealth made it possible to have an abortion soon was similar across pregnancy duration categories.

**Table 3:** Associations between patient characteristics and whether telehealth made it possible to obtain a timely abortion (N=1586).

Overall			Prevalence ratio (95% CI)	Marginal prevalence estimates (95% CI)
**Patient age (years) at abortion intake**				
	<25	1.4 (1.2-1.6)		48.3 (43.7-52.9)
	25-29	1.4 (1.1-1.6)		47.6 (42.8-52.4)
	30-34	1.1 (0.9-1.3)		38.8 (34.0-43.5)
	>34	Reference category		35.2 (30.0-40.4)
**Pregnancy duration at abortion intake (days)**				
	<35	Reference category		44.5 (39.9-49.1)
	35-49	0.9 (0.8-1.0)		40.6 (37.4-43.9)
	50-62	1.1 (0.9-1.3)		48.5 (42.0-55.0)
	≥63	1.1 (0.8-1.6)		50.0 (34.1-65.9)
**Food did not last in the last month**				
	No	Reference category		40.2 (37.4-42.9)
	Yes	1.3 (1.1-1.4)		51.2 (45.9-56.5)
**Race or ethnicity**				
	Asian, Native Hawaiian, or Pacific Islander	0.7 (0.5-1.0)		30.5 (21.7-39.3)
	Black	1.0 (0.9-1.3)		43.8 (35.8-51.9)
	Hispanic or Latinx	1.2 (1.0-1.4)		48.8 (41.9-55.6)
	White	Reference category		41.9 (38.6-45.3)
	Multiracial, other, or unknown	1.1 (1.0-1.3)		46.3 (40.7-52.0)
**Rural residence**				
	Urban	Reference category		41.7 (39.1-44.2)
	Suburban or rural	1.4 (1.2-1.6)		56.9 (48.9-65.0)
**Round-trip driving miles averted (miles)**				
	<5	1.2 (1.1-1.4)		47.7 (43.0-52.4)
	5-24	Reference category		39.3 (35.8-42.8)
	25-49	1.1 (0.9-1.3)		42.3 (34.7-49.9)
	50-99	0.9 (0.7-1.2)		35.0 (26.5-43.5)
	≥100	1.6 (1.3-1.9)		62.4 (52.9-71.8)

## Discussion

### Principal Findings

In this study, we found that direct-to-patient telehealth can be an important tool to overcome disparities in abortion access. While the expansion of telehealth is key to improving health equity broadly, abortion care is largely siloed to abortion facilities, which are few and far between and are now closing in record numbers [31]. Therefore, the impact of telehealth on improving equitable abortion access is even greater than for other health care services.

We found that using a telehealth abortion model can avert substantial driving distance and time for those with access to a car and travel time for those who would have used public transit. Our sample drew overwhelmingly from urban areas and from protected access states. Even so, while most of our sample resided close to an abortion facility, 15% lived 25 miles or further, and 7% lived 50 miles or further. Therefore, telehealth can expand the geographic reach of abortion services in states where abortion remains legal.

Telehealth abortion fits within an environmental justice framework, which views telehealth as a means of reducing the carbon footprint of health care services and space as a key component of health disparities [32,33]. Marginalized communities disproportionately bear the brunt of both unequal health policies and the unequal impacts of climate change. This analysis elucidates the connection between environmental justice and reproductive justice by examining abortion access within this framework of geospatial and environmental inequality.

These results suggest that telehealth abortion is poised to address health equity concerns across age, socioeconomic status, and geospatial location. Young people face unique logistical and privacy challenges, many of which can be alleviated by using telehealth, which allows patients to maintain privacy and avoid travel [34]. For low-income patients, the cost of abortion is often a substantial barrier to care, which is only exacerbated when coupled with the costs of associated travel [35]. Telehealth abortion has the potential to address geographic inequities in access to care for those living in abortion deserts or rural areas within protected access states. These findings on telehealth abortion fit within a broader literature that views telehealth as beneficial for reducing health care costs [36], serving underserved and rural patients [37,38], and easing the convenience and comfort of health care provision [39,40].

Compared to the national population of patients who obtained abortions during the same time period, patients in our sample who obtained abortions by telehealth were more likely to be older, White, and have higher socioeconomic statuses [41]. Thus, telehealth abortion may be reaching a subset of patients who are more resourced or have higher technology literacy. However, when patients were asked what would have happened had they not obtained the telehealth abortion, we found that for nearly half, telehealth made it possible to obtain timely abortion care. Telehealth was more likely to play this instrumental role in obtaining an abortion among patient populations who are known to face the most structural barriers to abortion care, such as younger people, those experiencing food insecurity, those residing in rural areas, and those who resided far from an abortion facility [2,42]. Telehealth was less likely to make timely abortion care possible for Asian patients than White patients, perhaps due to urban residence and proximity to abortion providers. Given that telehealth abortion care is situated within a highly unequal health care landscape, there is interest in its role in mitigating inequities in abortion access [43,44]. Our results suggest that telehealth is playing different roles among different demographic groups within our sample. More specifically, while currently, White, urban patients might prefer telehealth due to its convenience or privacy, many patients from groups facing structural barriers to abortion care, such as people of color, those living on lower incomes, those who are younger, and those residing in rural areas, face greater distances to abortion facilities and thus may rely on telehealth for abortion access. These findings suggest that telehealth is key to promoting equitable access to abortion care in the United States, and expanding access to telehealth would be key for these groups.

We found that for patients who resided more than 100 miles from an abortion facility, telehealth was more likely to make it possible to access timely abortion care. This finding echoes prior research that has documented that geospatial barriers can determine whether patients obtain wanted abortions [4,5]. These results bolster the need for alternatives to in-clinic abortion care, especially as geographic access to abortion facilities grows increasingly unequal.

### Limitations

This paper has several limitations. Our use of ZIP codes as the location of patient residences reduces the accuracy of the travel distances and travel times we calculated. This likely resulted in overestimated distances and times for some patients and underestimated distances and times for others; however, the net misclassification remains unknown [45]. Only one-fourth of patients elected to participate in the surveys, which may have introduced selection bias and limited the generalizability of these findings to the broader population of virtual clinic patients. Some patients from restricted access states who traveled for abortion care or used various mail-forwarding techniques may have chosen not to disclose their true home ZIP codes. Further, this analysis was conducted on data collected prior to the *Dobbs* decision in June 2022, after which 14 states have banned abortion care. Given the increased wait times for abortion care that have resulted from widespread abortion restrictions, many patients who seek in-person abortion care may need to travel further than their closest abortion facility. Therefore, our analysis may have underestimated the travel averted that we documented in this study. The overwhelming majority of patients in our sample resided in states that allow telehealth for abortion care. Most other states have laws that prohibit telehealth for abortion [15]. Our analysis, therefore, does not capture the full range of travel that could be averted if telehealth abortion care were available across the country. While we did not find that telehealth was more likely to make a critical difference in obtaining an abortion for people of color, this finding may be limited by the racial composition of our sample, in which Black and Hispanic patients, who face the greatest barriers to abortion care, were greatly underrepresented compared to the population of US patients who obtained abortions [41]. Despite these limitations, this study highlights the benefits of telehealth services by being among the first to use geospatial analysis to examine the travel averted, across both driving and public transit transportation, from using a telehealth model for abortion care.

### Conclusions

This paper makes important contributions to a growing body of work on telehealth abortion and the broader literature on telehealth. Our findings support the role of telehealth abortion in reducing travel distance, time, and costs, which in turn mitigates the inequities embedded in abortion provision based on geospatial location, socioeconomic status, and local abortion policy. We found that telehealth can play a key role in accessing an otherwise unobtainable or delayed abortion, especially for marginalized patient populations. Future research should examine how telehealth abortion services can be tailored to improve health equity and digital inclusion. These findings may help direct future policy on expanding access to telehealth abortion, especially in the wake of the *Dobbs* decision, which is continuing to exacerbate longstanding inequities in abortion access. While states that ban abortion are unlikely to support telehealth for abortion, there are 6 states that permit abortion but restrict telehealth abortion care [14]. States invested in health equity that want to safeguard access in an increasingly restricted abortion landscape should legalize telehealth abortion. There is a need for federal actions to protect patients who seek and providers who dispense telehealth abortion care across state lines.

## Appendix

Multimedia Appendix 1 Definition of primary outcome measure.

Multimedia Appendix 2 Density plot of travel averted by using telehealth.

## Abbreviations

CHAT: California Home Abortion by Telehealth Study
PP: prevalence percentage
PR: prevalence ratio
REDCap: Research Electronic Data Capture

## Data Availability

The data sets generated and analyzed during this study are not publicly available due to privacy concerns of patients who obtained abortions and the changing legal status of abortion care but are available from the corresponding author on reasonable request.
