# Supplementary figures and images for: The Role of Telehealth in Promoting Equitable Abortion Access in the United States: Spatial Analysis
Source: JMIR Public Health Surveill. 2023 Nov 7;9:e45671. doi: 10.2196/45671 (PMC10664017; doi:10.2196/45671)

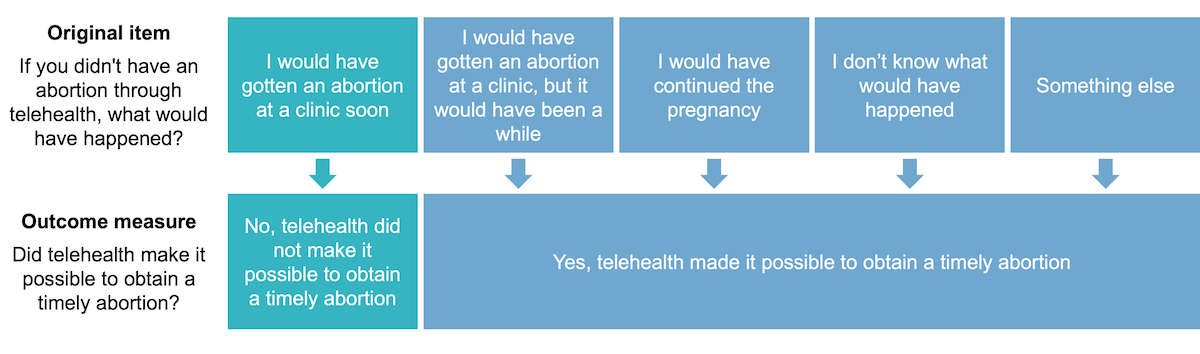

Supplement: Multimedia Appendix 1 [file publichealth_v9i1e45671_app1.png]

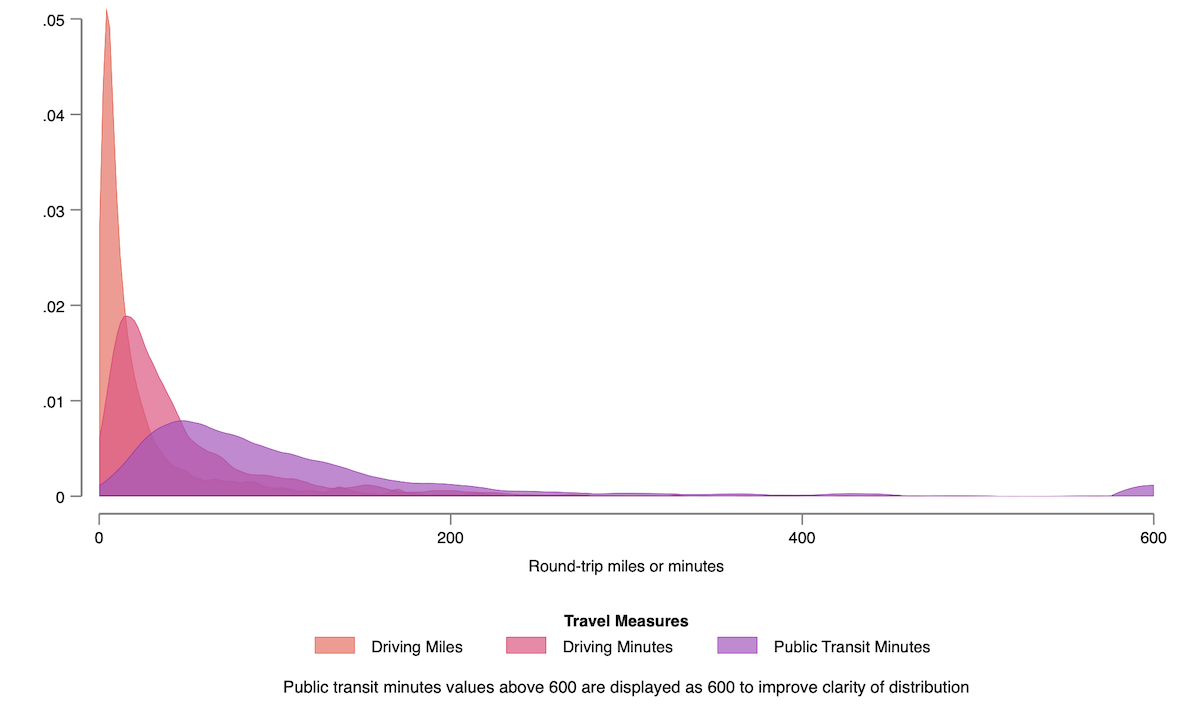

Supplement: Multimedia Appendix 2 [file publichealth_v9i1e45671_app2.png]
